# Controlling the orbital angular momentum of high harmonic vortices

**DOI:** 10.1038/ncomms14970

**Published:** 2017-04-05

**Authors:** Fanqi Kong, Chunmei Zhang, Frédéric Bouchard, Zhengyan Li, Graham G. Brown, Dong Hyuk Ko, T. J. Hammond, Ladan Arissian, Robert W. Boyd, Ebrahim Karimi, P. B. Corkum

**Affiliations:** 1 Department of Physics, University of Ottawa, 25 Templeton St, Ottawa, Ontario, Canada K1N 6N5; 2 Joint Attosecond Science Laboratory, University of Ottawa and National Research Council of Canada, 100 Sussex Drive, Ottawa, Ontario, Canada K1N 5A2; 3 The Institute of Optics, University of Rochester, Rochester, New York 14627, USA

## Abstract

Optical vortices, which carry orbital angular momentum (OAM), can be flexibly produced and measured with infrared and visible light. Their application is an important research topic for super-resolution imaging, optical communications and quantum optics. However, only a few methods can produce OAM beams in the extreme ultraviolet (XUV) or X-ray, and controlling the OAM on these beams remains challenging. Here we apply wave mixing to a tabletop high-harmonic source, as proposed in our previous work, and control the topological charge (OAM value) of XUV beams. Our technique enables us to produce first-order OAM beams with the smallest possible central intensity null at XUV wavelengths. This work opens a route for carrier-injected laser machining and lithography, which may reach nanometre or even angstrom resolution. Such a light source is also ideal for space communications, both in the classical and quantum regimes.

## Abstract (web summary)

Few methods can produce orbital angular momentum beams in the extreme ultraviolet or X-ray, and controlling orbital angular momentum on these beams remains challenging. Here the authors apply wave mixing to a tabletop high-harmonic source and control the topological charge of extreme ultraviolet beams.

Optical vortices^1^ have become an important research topic with extensive applications in particle trapping^2^, super-resolution imaging^3^, optical communications^4^ and quantum optics^5^,^6^. Such vortex beams can be flexibly produced, measured and manipulated with infrared and visible light^1^,^7^,^8^. For example, stimulated emission depletion (STED) microscopy overcomes the Abbe diffraction limit by using the dark centre of the first-order optical vortex beam with a helical wavefront characterized by e^*ilϕ*^, where *l* is the topological charge^1^,^3^. The resolution of STED microscopy can reach *λ*/100 when visible light is used^9^, where *λ* is the wavelength of the pump beam. Structured light sources at shorter wavelengths open a route to increasing the resolution still further. However, previous methods^10^,^11^,^12^,^13^ of producing short wavelength vortex beams via high-harmonic generation place topological charge on the fundamental beam, and therefore fail to produce high harmonics with low-order orbital angular momentum (OAM). The topological charge of the fundamental is multiplied by the harmonic order during harmonic generation^10^,^11^,^12^,^13^,^14^.

In this paper, we demonstrate a tabletop source that can generate extreme ultraviolet (XUV) beams with controllable topological charges. The generation process is driven by a Gaussian infrared laser beam that is perturbed by a weak beam of the same colour that carries OAM. A *q*-plate^8^ imprints OAM onto the perturbing infrared beam through geometric phase. Unlike other devices used in the previous methods^10^,^11^,^13^, when we select a single polarization, a *q*-plate eliminates the mode contamination caused by dispersion^15^. By varying the topological charge of the perturbing beam, we control the OAM of the diffracted radiation, enabling us to achieve *l*=±1 high-harmonic beams. We also develop a holographic technique to measure the OAM of any harmonic. For a reference, we use a separate and highly divergent coherent source. The interference between the reference and the OAM beams determines the spiral wavefronts of XUV beams. By creating *l*=+1 beam for any harmonics, this experiment provides a crucial step towards applications of structured XUV beams. These vortex beams can be focused down to nanometre scale^16^ and they open a route for carrier-injected laser machining and lithography^17^,^18^ to reach STED-like super resolution, which we will discuss in the concluding paragraph of this paper.

## Results

### Generating high-harmonic vortex beams with *l*=±1

**Figure 1:**
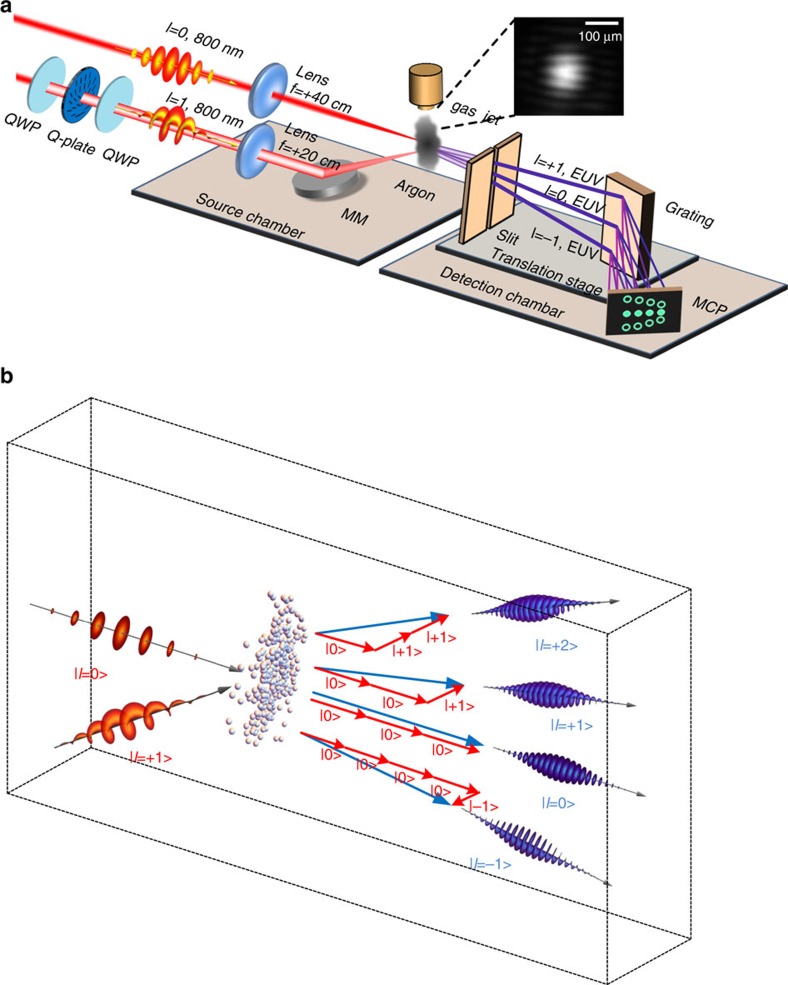
Experimental set-up for generating XUV beams with arbitrary OAM. (**a**) A strong infrared laser beam is overlapped with a weak infrared vortex beam in the argon–gas interaction medium. Their interference creates an intensity and phase distribution with a forked structure in the gas medium. The distribution is transferred to the phase of the emitting dipoles. The produced XUV is diffracted by this forked-grating structure. The three diffracted high-harmonic XUV beamlets that are produced carry +1, 0 and −1 units of OAM. The XUV beams are spectrally resolved in a detection chamber and detected by a microchannel plate. The slit and the grating can be translated together to reconstruct the beam profile. QWP, quarter wave plate; MCP, microchannel plate; MM, motorized mirror. Inset: the interference pattern between the Gaussian driving beam and the vortex beam at the focal plane. This distribution induces the fork-shaped phase grating of the emitting dipole. (**b**) Illustration of the conservation of linear and OAM in a third-order harmonics generation case. The emitted angle of the XUV photon is determined by vector addition of linear momentum. The topological charge of the XUV photon equals the sum of the topological charges of all the absorbed infrared photons.

Figure 1a illustrates the experimental arrangement used to generate high-harmonic beams with OAM. A linearly polarized 800-nm infrared Gaussian beam is focused to an intensity of 2 × 10^14^ W cm^−2^ in a noble-gas medium, while another weaker beam with the same polarization and the same colour is given one unit of OAM by passing it through a *q*-plate before focusing it to an intensity of 2 × 10^12^ W cm^−2^. These two beams overlap in both time and space in the nonlinear medium at a relative angle of 40 mrad. The strong Gaussian beam drives the high-harmonic generation, while the weak vortex beam controls the process. We use a thin noble-gas medium (see ‘Methods' section) to minimize propagation effects^19^. The different orders of harmonics are then spectrally resolved in the horizontal direction by a diffraction grating, and recorded by a microchannel plate as described in the ‘Methods' section. We move the slit and the diffraction grating together to reconstruct the spatial profile of each harmonic (see Supplementary Note 1).

The two infrared beams are both linearly polarized and interfere in the gas medium, as shown in Fig. 1a. Since the weak vortex beam has a helical wavefront, the overlapping infrared beams form a fork-shaped electric field distribution. In contrast to the model with second harmonic perturbation^11^, by using the same colour wave mixing scheme, we form a static holographic pattern in both its intensity and phase. Such a fork-shaped distribution imprinted on the driving field alters the electron trajectories, which consequently modifies the phase of the dipole emission. This distribution induces a weak grating-like spatial phase variation in the emitting dipoles, thereby causing the XUV beam to diffract in the vertical direction. The first-order diffracted beams should carry the same OAM as the perturbing beam. This prediction is based on the semi-classical model of high-harmonic generation in the strong-field limit^20^ and can be understood qualitatively. The phase modulations appear directly on the XUV beam, while the amplitude modulation is transferred to phase through the influence of amplitude changes to the re-collision electron phase and therefore to the induced dipole that is emitted^21^.

An analogy with holography may be helpful. It is possible to think of the vortex XUV beam as being generated in a holographic fashion: the driving Gaussian beam and weak vortex beam, respectively, serve the roles of a reference beam and an object beam. Their interference imposes a fork-shaped holographic structure onto the plane of the emitting dipoles. As in standard holography, the diffracted orders preserve the wavefront of the incident object (vortex) beam. As the analogy with holography implies, any method that can induce phase modulation in the dipole emission, including a forked-grating pattern in the alignment distribution of multi-electron molecules^22^, can be used to imprint such holographic phase pattern in the gas medium.

As is often the case in nonlinear optics, momentum conservation gives a useful complementary perspective. Figure 1b depicts the conservation of both the linear and the OAM of the absorbed infrared photons, in the simplest case of third-harmonic generation. The emitted angle of the XUV photon is determined by the vector addition of linear momentum^23^. For this reason, the diffraction order in the vertical direction indicates how many photons from the vortex beam are absorbed. If OAM is also conserved, as shown in equation (1),


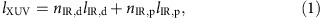


where *l*_XUV_ is the topological charge of the emitted XUV photon, *n*_IR,d_ and *n*_IR,p_ are the number of photons absorbed from the driving and perturbing beams, respectively, *l*_IR,d_ and *l*_IR,p_ are the topological charges of the driving and perturbing beams, respectively. Then the topological charge of the XUV photon *l*_XUV_ must equal the sum of the topological charges of all the absorbed infrared photons, as predicated in ref. 11. Because photons from the strong driving field carry zero OAM (*l*_IR,d_=0), and the ±1st order absorbs or emits one photon from the weak vortex beam (*n*_IR,p_=±1, *l*_IR,p_=1), any harmonic in the ±1st order diffracted spectrum should have ±1 unit of OAM. In other words, the topological charges of the XUV beams are determined by the perturbing infrared beam.

**Figure 2:**
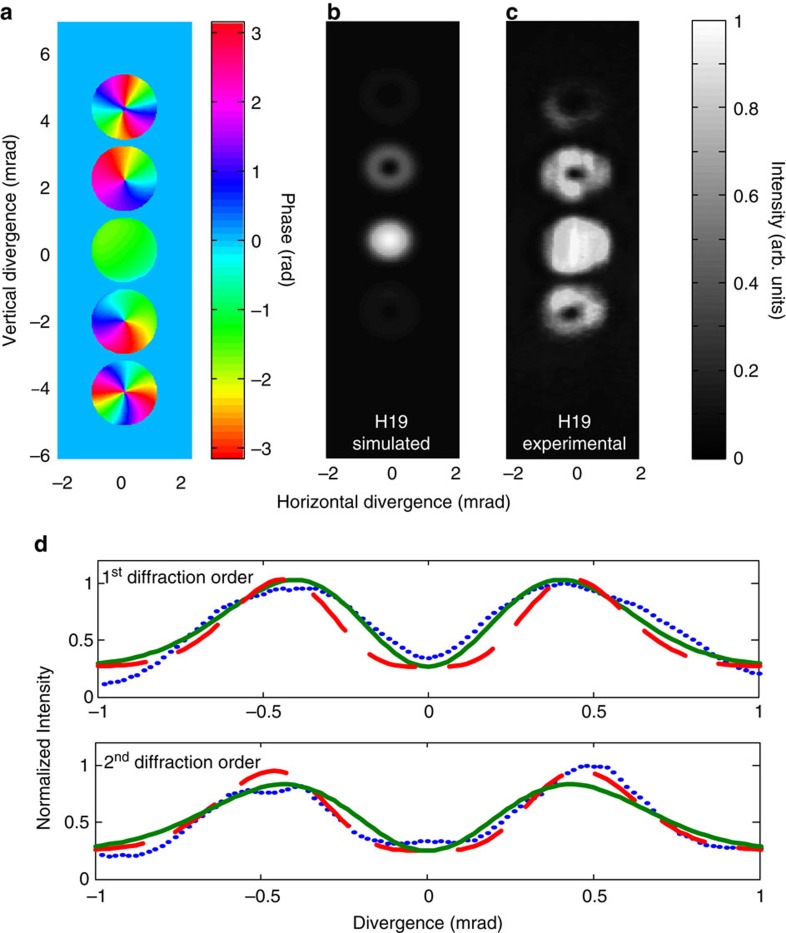
Phase and intensity profiles of the high-harmonic vortex beams with controlled OAM. (**a**,**b**) Simulated results of phase and intensity profiles of the 19th harmonic emission in the far field. (**c**) Experimental results of the intensity profile of the 19th harmonics recorded on the microchannel plate. (**d**) Intensity fittings of 1st and 2nd diffracted beam intensity profiles with 1st and 2nd order Laguerre polynomials. The measured beam profile for 1st diffracted order (blue dotted line) fits the green solid line (*l*_XUV_=1), and the measured beam profile for 2nd diffracted order (blue dotted line) fits the red dashed line (*l*_XUV_=2). This is an indication of the absolute value of the topological charges.

Using the strong-field approximation^24^ for a single plane of emitters, we obtain the simulated results in the far field that we show in Fig. 2a,b. The figure shows that the beams diffracted in first-order exhibit donut shapes in their intensity profiles and have spiral wavefronts changing from 0 to 2*π* in the phase plot.

As we increase the energy in the perturbing vortex beam, the depth of the phase modulation increase and a second diffraction order appears. It also has a donut-shaped intensity profile, but is less intense than the first-order beams. When the perturbing field has an intensity of ∼10^−2^ to the driving field, the energy ratio between the +1 diffraction order and the zero order at the 17th harmonics is 0.18. The diffraction efficiency depends on the intensity of the perturbing beam and the mode overlap between the perturbing and driving beams. Experimental results, including second-order diffraction, are shown in Fig. 2c for the 19th harmonics and see Supplementary Fig. 2 for the 17th harmonics. Since the process is perturbed by a same colour 800 nm beam, harmonic 18th is missing due to the half-cycle symmetry of the electric field^21^.

The second-order diffracted beams have absorbed two photons from the perturbing vortex beams and from the conservation of OAM should therefore carry two units of OAM. These double-helical wavefronts with an azimuthal phase that varies from 0 to 4*π* are also confirmed in our model simulations. In the experimental result in Fig. 2c, we see that the second-order beams have a larger hole in their centre. The faster azimuthal phase variation of the *l*_XUV_=2 beam causes radiation to diffract from the singular centre more rapidly. The intensity profile of each beam is highlighted in Fig. 2d for both the first- and second-order beams. The larger dark centre of the *l*_XUV_=2 beam fits the second-order polynomial much better than it does a first-order (*l*_XUV_=1) beam profile.

### Interferometric characterization of the helical wavefronts

**Figure 3:**
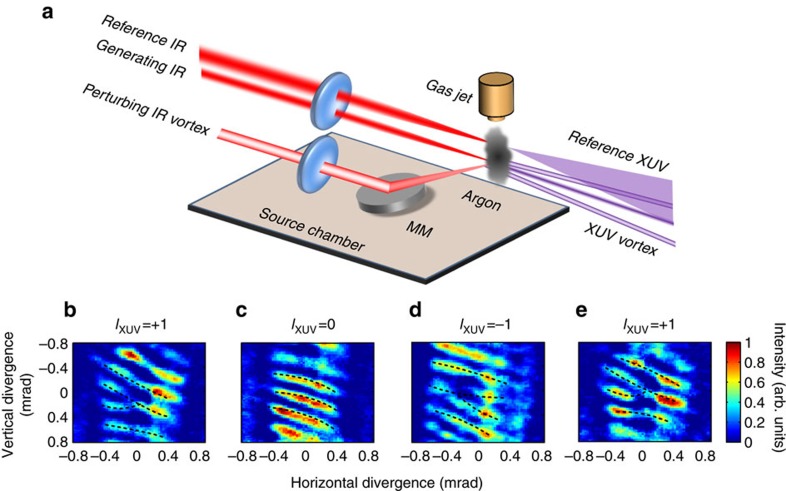
Characterization of wavefronts and modulation of topological charges. (**a**) Schematic of the interferometric wavefront characterization procedure. (**b**,**c**,**d**) Interference patterns of the Gaussian reference beams and OAM beams of charges *l*_XUV_=+1, 0, −1. (**e**) The sign of topological charge at −1st order diffraction is flipped from *l*_XUV_=−1 to *l*_XUV_=+1 by changing the incident polarization.

We use interferometry to characterize the wavefront and the direction of the helices. The experimental set-up for measuring the phase structure is shown in Fig. 3a. A third coherent infrared driving beam is now added to the experiment to produce a reference XUV beam. After passing through the spectrograph, the harmonics from the two sources interfere on the microchannel plate. Figure 3b–d shows the interference patterns between the reference XUV beams and diffracted XUV vortex beams. For the +1st order diffracted beam, the forked pattern has one more fringe on the left than on the right, labelled by dashed line in Fig. 3b. In contrast, the interference of the reference beam with the −1st order (Fig. 3d) shows one additional fringe on the right. This difference in the fringe pattern indicates the opposite handedness of the helical wavefronts.

The approach of using a weak field to control the harmonics created by the strong fundamental pulse opens an important channel to link mature infrared optical devices to XUV radiation, and can serve as an alternative solution to making optical components that function in the XUV and soft X-ray region. Since the perturbing beam is more than two orders of magnitude weaker than the driving laser, this method allows liquid–crystal-based, programmable optical devices to be used in spite of their rather low-damage thresholds. This brings us to X-ray-based communications, which is the first of the two important applications that we propose.

The sign of the topological charge on any diffracted harmonic can be changed by changing the linear polarization of the control beam that is incident on a *q*-plate^8^, and the incident polarization can be modulated at MHz rates with a polarization modulator such as a Pockels cell^15^. We demonstrate this control by rotating a wave plate to change the topological charge from *l*_XUV_=+1 to *l*_XUV_=−1 as shown in Fig. 3d,e. For applications in super-dense coding^4^,^25^ and quantum communication^26^, one can even use cascaded Pockels cells and *q*-plates of various topological charges^27^, with each Pockels cell operating at MHz rates, consistent with the MHz-repetition-rate, high-harmonic sources that have already been demonstrated with both cavity-based and single-pass schemes^28^,^29^,^30^.

## Discussion

The potential for fast modulation makes high-harmonic beams potential candidates for long-distance space communications, since shorter-wavelength beams diffract much less on propagation and support extremely large data transmission bandwidths. X-ray-based communication may also overcome ionization blackouts and make it possible to communicate with hypersonic vehicles or re-entering spacecraft. But this is not all: there is a rapidly increasing interest in using the entanglement of OAM in quantum key distribution and communication^6^,^27^. Using classical concepts, we show in the Supplementary Note 2 the potential for interesting quantum optics with high-harmonic generation.

The second important application that we propose here is super-resolution laser machining. The essence of STED microscopy is to use the saturation of depletion to exploit the singularity at the centre of an OAM beam^3^. This STED idea can be used for other phenomena that have a similar response. In laser processing, for example, desorption of a thin layer^31^ can potentially be exploited in a STED-like manner. Consider the case where a donut-shaped XUV beam is linearly absorbed by a thin layer of polymer deposited on a fused silica substrate. XUV-injected electron–hole pairs are created in the thin-layer material except at the dark centre of the beam. A following infrared laser pulse can cause an avalanche build-up of these carriers, depositing energy in the material where the carriers were created. The intensity of guiding pulse can be much lower than the damage threshold because the following infrared pulse^17^ deposits any extra energy that is required. The thin layer will be desorbed where the deposited energy exceeds the damage threshold. Since XUV beams are selectively absorbed near the surface and the absorption can be tuned to exploit the atomic structure of the layer or substrate, the machining stops at the thin film/substrate interface when the thin layer is completely removed. Such selective absorption prevents the substrate from being engraved and gives rise to the saturable behaviour that enables super-resolution processing.

The short wavelength of XUV beams affords much higher resolution than the machining process based on the use of an infrared field. Therefore, we propose that carrier-injection-controlled machining, which so far has only been explored with Gaussian control beams at ultraviolet wavelength^17^, will now enable laser machining (and lithography) on the sub-nanometre scale.

## Methods

### Generation of high-harmonic beams with controlled OAM

The driving field in the experiment is created by interfering two beams. A strong infrared Gaussian beam with wavelength of 800 nm, pulse duration of 30 fs and energy of 500 μJ is focused into an argon gas by means of a 40 cm lens. In addition, a weak perturbing vortex beam with wavelength of 800 nm, pulse duration of 50 fs and energy of 250 μJ is focused by a 20 cm lens. The weak vortex beam is formed by a *q*-plate—a liquid crystal device that can imprint OAM onto infrared beams.

The centre wavelength of the *q*-plate is tuned to 800 nm and the *q*-plate is designed to give the transmitted beam a topological charge of one. The two non-collinear beams overlap in both time and space. Their 40 mrad relative angle ensures that the diffracted XUV beams are also spatially separated. Since the OAM of a light depends on the axis of the beam, a motorized mirror ensures good spatial overlap of the two beam centres.

Argon gas is used as the nonlinear medium. The gas is injected through a 200-μm nozzle into the vacuum chamber via a synchronized Even Lavie gas jet at a 500 Hz repetition rate. The backing pressure in the chamber reaches 10^−4^ mbar.

The harmonic beam propagates to a spectrometer in a differentially pumped detection chamber. The spectrometer consists of a slit, a grating and a microchannel plate. The slit and grating are translated together to reconstruct the beam profiles of the high harmonics. The beams are then spectrally resolved in the horizontal direction by an 87° grazing-incidence grating with 1,200 lines per mm, and are then detected by a microchannel plate. A camera records the fluorescent screen at the back of the microchannel plate. On the spectrogram, different positions along the horizontal direction correspond to different photon energies, or alternatively, to the order of the harmonics.

When characterizing the wavefront by means of interferometry, a third infrared beam is added to produce XUV reference beams. The third beam has a larger beam diameter before the focusing lens, which leads to a tighter focus than the focus of the generating infrared beam. The divergence of the reference beam is large enough to enable the reference XUV beams to overlap the +1st, 0th and −1st orders XUV vortex beams. The two generating sources are separated by 150 μm, which is far enough to eliminate crosstalk between the two XUV sources.

The detection set-up in the interferometric measurement for the holographic measurement does not contain a slit. This allows the entire beam to be observed. To allow both spatial and spectral resolution, the microchannel plate is placed close enough to the grating that phase front information is preserved.

### Strong-field approximation simulation with single-plane emitters

Since the gas medium is only 200 μm thick, we simulate the generation process with emitters in a single plane, without propagation effects. Each emitter is driven by the local time-dependent electric field created by the interference of the strong Gaussian beam and the weak vortex beam. The strong-field approximation dipole moment in 2D is calculated pointwise at the focal plane. It is then decomposed in the Fourier domain to obtain near-field emission of each harmonic order. The far-field Fraunhofer diffraction image is obtained by performing 2D Fourier transformation.

### Data availability

The data that support the findings of this study are available from the corresponding author on reasonable request.

## Additional information

**How to cite this article:** Kong, F. *et al*. Controlling the orbital angular momentum of high harmonic vortices. *Nat. Commun.* **8**, 14970 doi: 10.1038/ncomms14970 (2017).

**Publisher's note:** Springer Nature remains neutral with regard to jurisdictional claims in published maps and institutional affiliations.

## Supplementary Material

Supplementary Information Supplementary Figures, Supplementary Notes and Supplementary References
