# Supplementary material for: Controlling the orbital angular momentum of high harmonic vortices
Source: Nat Commun. 2017 Apr 5;8:14970. doi: 10.1038/ncomms14970 (PMC5382317; doi:10.1038/ncomms14970)
Supplement: Supplementary Information — Supplementary Figures, Supplementary Notes and Supplementary References [file ncomms14970-s1.pdf]

**Supplementary Note 1**

**Reconstructing the intensity profiles of diffracted XUV vortex beams.** The generated harmonics propagate to the far field and are detected by an imaging spectrometer. The spectrometer is composed of a vertical slit, a 1200 lines/mm grating, a micro-channel plate, and a CCD camera; the plane of the slit is imaged onto the micro-channel plate using the imaging grating, and the CCD camera records the intensity profile on the phosphor screen of the micro-channel plate. The grating separates different orders of harmonics by mapping wavelength to the horizontal axis of the micro-channel plate.

The harmonics beams are truncated vertically when passing through the slit of the spectrometer, as shown the yellow boxes in Supplementary Figure 1. In order to measure the full transverse profile of each harmonic, the slit and grating are moved simultaneously by a translation stage. Multiple images are recorded when the stage is moving and are composed according to different stage positions. The vertical slit is chosen to have a width of 100μm, which ensures both sufficient flux and horizontal resolution.

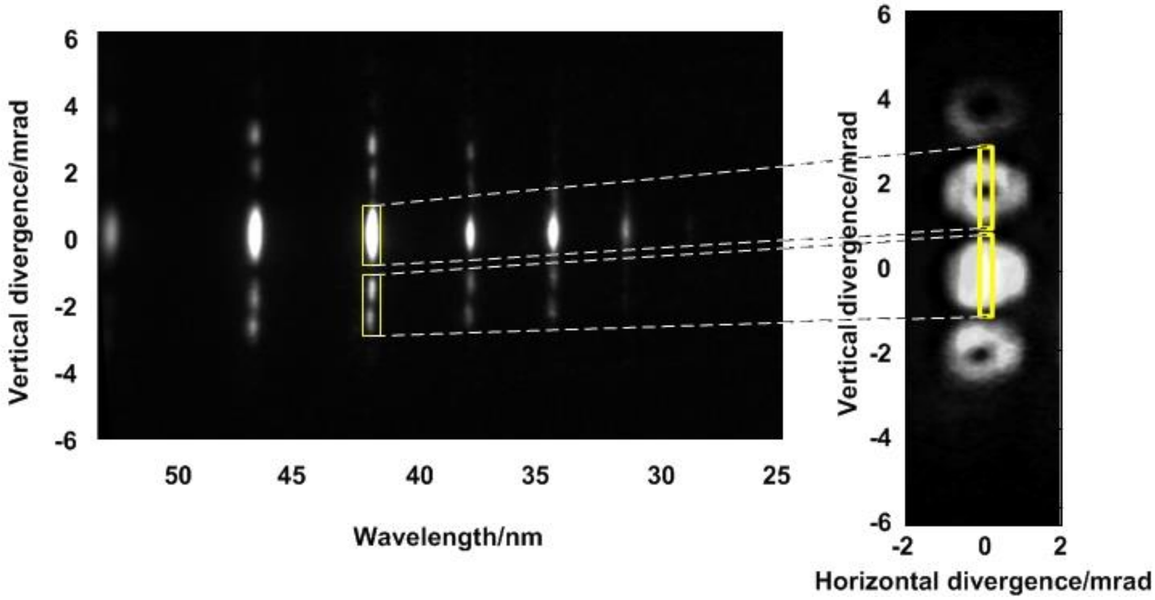


**Supplementary Figure 1 Reconstructing the intensity profiles of diffracted XUV vortex beams.** Left: the image recoded directly by the camera from the phosphor screen. The large spots around 0 mrad corresponds to the zeroth order Gaussian beams ($l_{\mathrm{XUV}}$=0 modes), while the split spots above and below corresponds to the diffracted XUV vortex; Right: reconstructed image by horizontally translating the slit and the grazing incident grating.

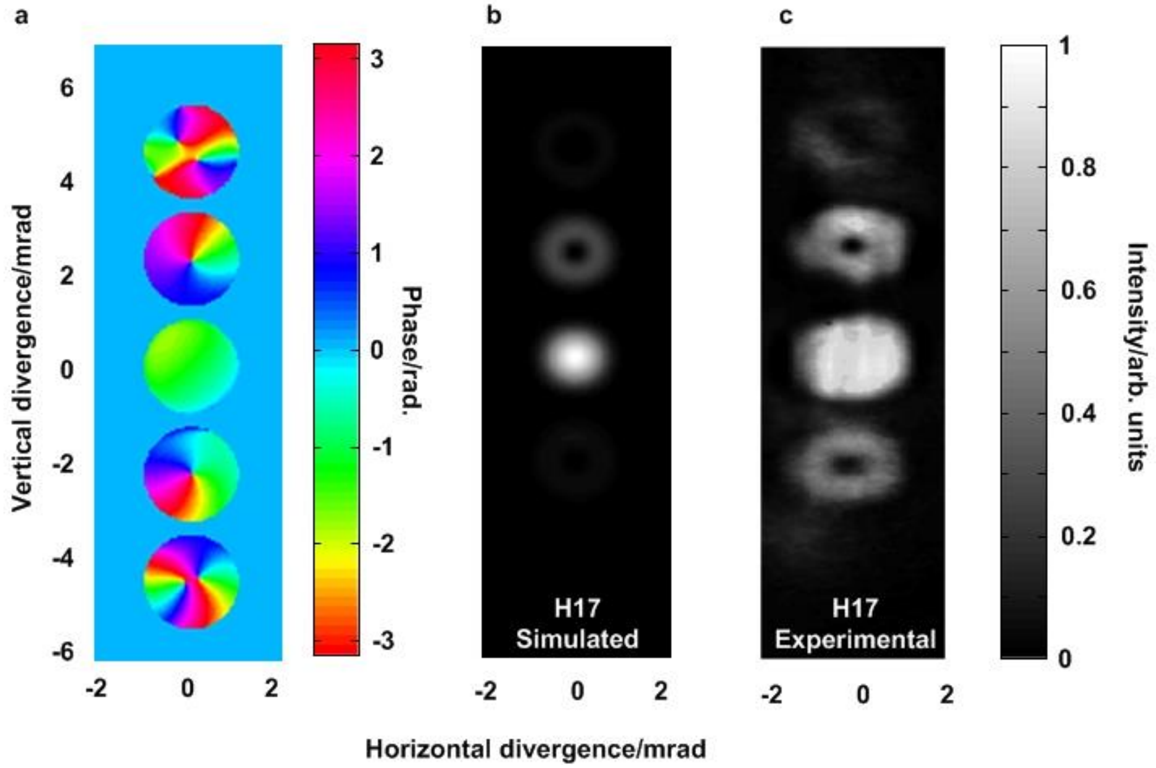


**Supplementary Figure 2 Phase and intensity profiles of the 17th order high-harmonic vortex beams with controlled OAM. a, b** Simulated results of phase and intensity profiles of the 17th harmonic emission in the far field. **c**, Experimental results of the intensity profile of the 17th harmonics recorded on the micro-channel plate.

**Supplementary Note 2**

**Classical estimate of the diffracted efficiency of the dipole phase grating – illustration of the potential for quantum optics of extreme nonlinear interactions.**

We make the following assumptions to describe the interaction of a single perturbing photon.

- We consider a single perturbing photon in a mode that optimally overlaps in space ($S$) and time ($t$) the driving laser pulse.
- We use $E=h\nu$ to estimate a classical energy of a single photon.
- We estimate the classical intensity by $E/St$.
- We use the known characteristics of diffraction gratings to estimate the diffracted light, although the diffraction efficiency is insufficient to describe the behavior of a single photon at specific frequency.

Our intension with the estimate below is to motivate the requirement for a quantum theory of extreme nonlinear optics. We are motivated by the potential to use one photon of an entangled pair as the perturbation beam. Of course, quantum optics can be important under much less extreme circumstances[1].

For our estimate, we will use an 800nm fundamental pulse with 1mJ energy per pulse or $N$=4×10$^{15}$ photons per pulse, 5×10$^{14}$ W cm$^{-2}$ intensity at focus and 10$^{-4}$ energy conversion to the sum of all harmonics in neon gas. For convenience we will assume that 100eV (65$^{\text{th}}$ harmonic) can represent the properties of all diffracted photons. These choices define the threshold diffraction efficiency $\eta_{\text{TXUV}}$=1.67×10$^{-10}$ that we will need for the grating to diffract one photon.

The diffraction efficiency of a sinusoidal phase grating is given by,

$$\eta_{\text{XUV}} = \left( \frac{J_1\left(\delta_{\text{XUV}}\right)}{J_0\left(\delta_{\text{XUV}}\right)} \right)^2 \underset{\delta\to 0}{=} \frac{1}{4}\delta_{\text{XUV}}{}^2 , \tag{1}$$

where $\delta_{\text{XUV}}$ is the phase modulation depth of the emission dipole at XUV wavelength in near field. Using equation (1.1) we calculate the threshold phase modulation depth for XUV

$$\delta_{\text{TXUV}} = \sqrt{4\eta_{\text{TXUV}}} = 2.6\times10^{-5}\,rad\ . \tag{2}$$

Knowing the required modulation, we can estimate the energy ratio between the strong and weak IR beams needed to create it. We define a perturbation parameter $\varepsilon = E_{\text{p}}/E_{\text{d}}$, where $E_{\text{p}}$ and $E_{\text{d}}$ are the perturbing and driving electric field respectively.

The phase estimate can be broken into two parts: a. the phase modification due to changes of the electron trajectory caused by amplitude variation of the driving laser field; b. the phase modification due to change of recombination time due to the phase variation of the driving field.

a. For the nonlinear phase that arises due to changes to the electron trajectory, the unperturbed phase of $q^{\text{th}}$ harmonics at frequency $\Omega$ is[2]

$$\Phi_{\text{amp}}\left(\Omega\right) = \int_{t_{\text{b}}}^{t_{\text{c}}} \frac{1}{2}\left(p-\mathbf{A}\right)^2 + I_{\text{p}}dt - \Omega t_{\text{c}}\ . \tag{3}$$

The change of action caused by a perturbed field can be evaluated by

$$\begin{aligned}\Delta\Omega_{\mathrm{amp}}(\Omega) &= \frac{\partial\Phi}{\partial t_{\mathrm{c}}}dt_{\mathrm{c}} + \frac{\partial\Phi}{\partial t_{\mathrm{b}}}dt_{\mathrm{b}} + \frac{\partial\Phi}{\partial p}dp + \int_{t_{\mathrm{b}}}^{t_{\mathrm{c}}}(p-\mathbf{A})\,dA\,dt \\ &= -I_{\mathrm{p}}dt_{\mathrm{b}} + \int_{t_{\mathrm{b}}}^{t_{\mathrm{c}}}(p-\mathbf{A})\mathbf{A}_{\mathrm{p}}dt \qquad (4) \\ &= \varepsilon\int_{t_{\mathrm{b}}}^{t_{\mathrm{c}}} -\frac{E_0}{\omega^2}E_0\left[\sin(\omega t_{\mathrm{b}}) - \sin(\omega t)\right]\sin(\omega t + \Delta\varphi)dt - I_{\mathrm{p}}dt_{\mathrm{b}}\end{aligned}$$

where $t_{\mathrm{c}}$ is time of recombination and $t_{\mathrm{b}}$ is time of birth, $p$ is the canonical momentum, $I_{\mathrm{p}}$ is the ionization potential of the gas medium, $A$ is the unperturbed vector potential of the driving laser field, $A_{\mathrm{p}}$ is the vector potential of the perturbing laser field, $E_0$ is the amplitude of the driving laser field, $\omega$ is the frequency of driving laser field and $\Delta\varphi$ is the phase difference between the driving and perturbing laser fields.

b. For the nonlinear phase influenced by the phase variation of the driving field,

$$\Delta\Phi_{\mathrm{ph}}(\Omega) = q\arctan\frac{\varepsilon\sin\Delta\phi}{1+\varepsilon\cos\Delta\phi}\ . \qquad (5)$$

The total phase shift of the dipole emission equals to the sum of the above two contributions. Since the perturbation is small we only keep the first order term. The coefficient dδ/dε is plotting in Supplementary Figure 3. The phase modulation is stronger for electrons which recombined later (the higher order harmonics).

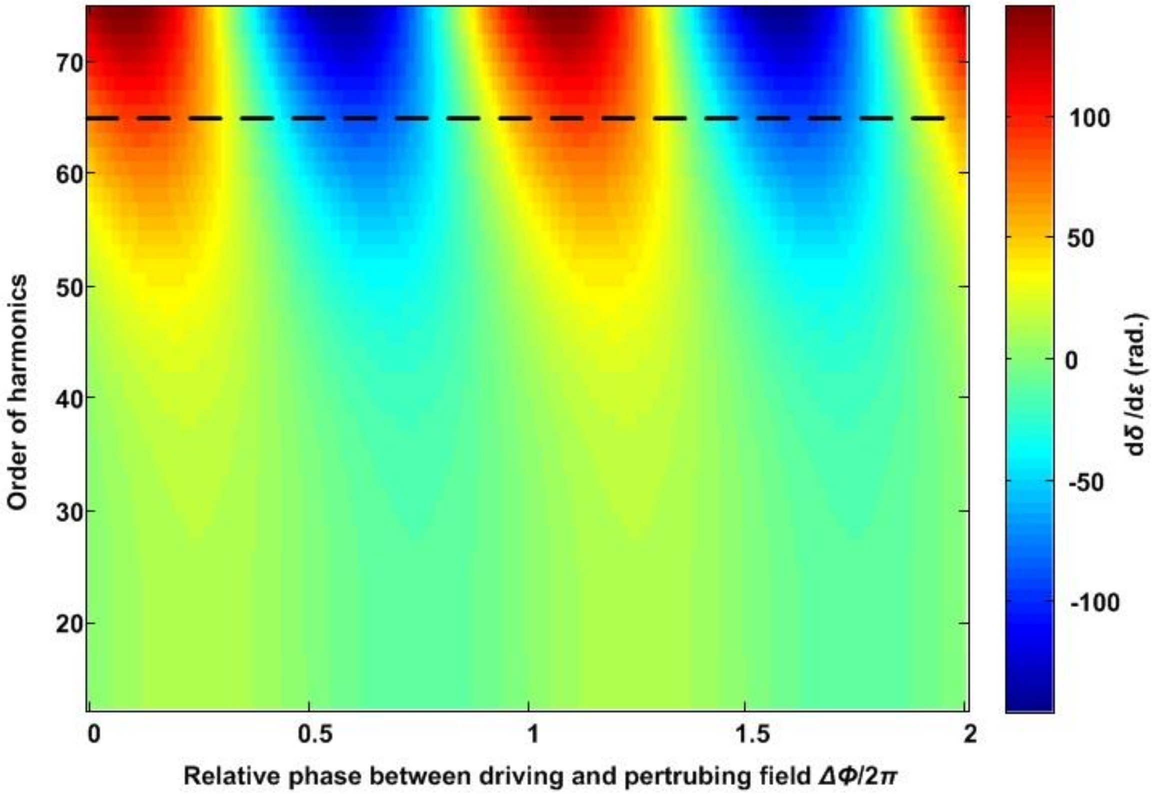


**Supplementary Figure 3 Phase modulation coefficient dδ/dε in radians for different electron trajectories**

Taking the trajectory corresponding to the 65th order (the dashed line), we can find that the ratio between modulation depth (d$\delta$) and the perturbation parameter (d$\varepsilon$) is 95. Therefore the ratio between the weak and strong IR field is

$$\varepsilon = \frac{d\varepsilon}{d\delta}\delta_{\mathrm{TXUV}} \approx 2.7\times10^{-7}, \quad (6)$$

and the minimum number of photons in the weak beam to deflect one XUV photon into the first order is

$$N_{\mathrm{p}} = N\varepsilon^2 \approx 290. \quad (7)$$

This implies that one high harmonic photon would be scattered for each 290 incident photons.

A similar diffraction efficiency is found by simulations using the strong field approximation. We plot the intensity ratio between the 1st and 0th order diffraction on XUV verse the number of photons in the weak pulse. Supplementary Figure 4 shows a linear dependence of the diffracted light intensity on the intensity of the weak control beam. This is the same scaling that we have discussed above. Projected to low intensity, the simulation results arrive at the same XUV diffraction efficiency $\eta_{\mathrm{xuv}}$ calculated above.

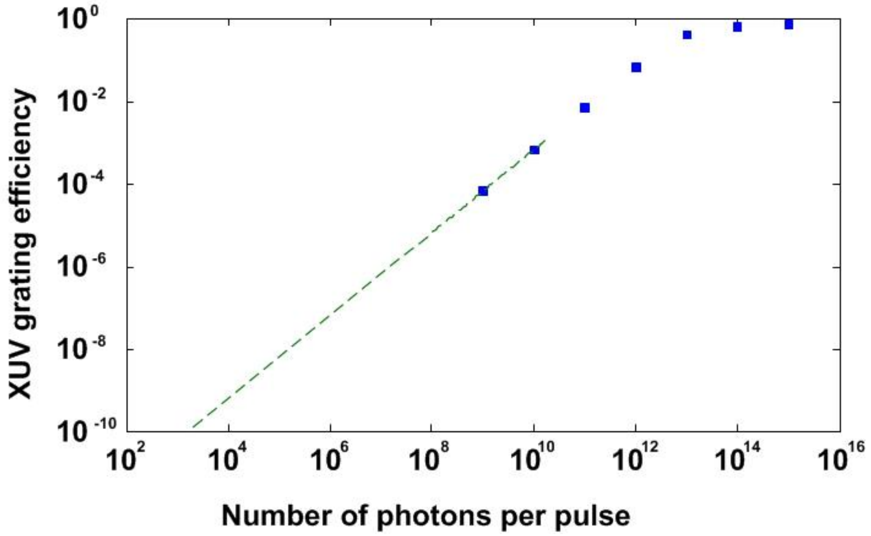


**Supplementary Figure 4 Scaling XUV diffraction efficiency verse the number of photons per weak pulse**

## Supplementary References


1. Straupe, S. & Kulik, S. QUANTUM OPTICS The quest for higher dimensionality. *Nat. Photon.* **4**, 585-586, (2010).
2. Lewenstein, M., Balcou, P., Ivanov, M. Y., L'huillier, A. & Corkum, P. B. Theory of High-Harmonic Generation by Low-Frequency Laser Fields. *Phys. Rev. A* **49**, 2117-2132, (1994).
